# ROS-induced Oxidative Injury involved in Pathogenesis of Fungal Keratitis via p38 MAPK Activation

**DOI:** 10.1038/s41598-017-09636-w

**Published:** 2017-09-05

**Authors:** Xia Hua, Wei Chi, Long Su, Jin Li, Zongduan Zhang, Xiaoyong Yuan

**Affiliations:** 1 Department of Ophthalmology, Tianjin Orbit Research Institute, the Second Hospital of Tianjin Medical University, Tianjin, China; 2 State Key Laboratory of Ophthalmology, Zhongshan Ophthalmic Center, Sun Yat-sen University, Guangzhou, China; 3 Zhejiang Eye Hospital, School of Optometry and Ophthalmology, Wenzhou Medical University, Wenzhou, China; 4 Tianjin Eye Hospital, Tianjin Key Lab of Ophthalmology and Visual Science, Clinical College of Ophthalmology, Tianjin Medical University, Tianjin, China

## Abstract

This study was to explore the mechanism by which reactive oxygen species (ROS)-induced oxidative stress involved in the pathogenesis of fungal keratitis using an *in vivo* experimental keratitis mouse model and an *in vitro* culture model of human corneal epithelial cells (HCECs). Compared to normal control mice and HCECs, ROS production was markedly increased in fungal corneas and HCECs exposed to *Candida albicans*, accompanied by p38 mitogen-activated protein kinases (MAPK) activation. Increased products of oxidative markers, malondialdehyde (MDA), 4–hydroxynonenal (HNE), mitochondria DNA 8-OHdG and aconitase-2 were observed in fungal infected corneas and HCECs. Fungal infection also increased the mRNA expression and protein production of heme oxygenase-1 (HMOX1) and cyclooxygenase-2 (COX2), with suppressed levels of antioxidant enzymes, superoxide dismutase-1 (SOD1), glutathione peroxidase-1 (GPx1) and peroxiredoxin-4 (PRDX4). Interestingly, the levels of ROS, oxidative markers and oxygenases were significantly reduced by co-cultured p38 inhibitor SB203580. Furthermore, SB203580 restored the levels of antioxidant enzymes suppressed by fungus. Our findings demonstrated for the first time that ROS-induced oxidative injury is involved in pathogenesis of fungal keratitis via p38 MAPK pathway, suggesting the novel therapeutic targets for the potential treatment of fungal keratitis.

## Introduction

Fungal keratitis is one of the leading causes of corneal blindness in developing countries^1^, and its incidence is increasing in highly populous countries in Asia and Africa^2, 3^. *Candida (C.) albicans* is a common fungus that can cause systemic mycosis, opportunistic mucosal candidiasis, endogenous endophthalmitis, and suppurative keratitis^4–7^. *C. albicans* specifically overcomes the ocular surface defense and penetrates the corneal stroma after converting into a filamentous form^8^. Hyphal invasion then induces host responses that may cause corneal inflammation and ulceration^9^, which in turn participates in the pathophysiology of fungal keratitis.

Oxidative stress caused by overproduction of reactive oxygen species (ROS) has been recognized to be an important factor in the pathogenesis of ocular surface diseases^10–12^. Under physiological conditions, ROS are generated by the mitochondrial electron transport chain and plays an important role in activating cellular signaling for survival. ROS overproduction may cause oxidative damage to the cell, which includes at least three reactions: lipid peroxidation of the membrane, intracellular oxidative modification of proteins, and oxidative damage to DNA^13–15^. The oxidative damage has been found to be mediated by increased expression of oxygenases and reduced production of antioxidant enzymes^16^.

Activation of mitogen-activated protein kinases (MAPKs) plays an important role in multiple physiological and pathological conditions^17^. The signaling pathway of one of the MAPK family members, p38 MAPK, has been found to be involved in ROS generation in different models of cell death^18, 19^, and a possible relationship between MAPKs and ROS has also been noted^20, 21^. However, it is still not clear whether and how fungal pathogens induce oxidative injury in keratitis and in human corneal epithelial cells (HCECs).

The present study explored corneal oxidative damage and the imbalance between oxidative and antioxidant enzymes in a mouse model of fungal keratitis as well as in a culture model of primary HCECs exposed to *C. albicans*, and our data reveal a potential mechanism by which ROS can induce oxidative stress via p38 MAPK activation.

## Results

### Experimental fungal keratitis

**Figure 1:**
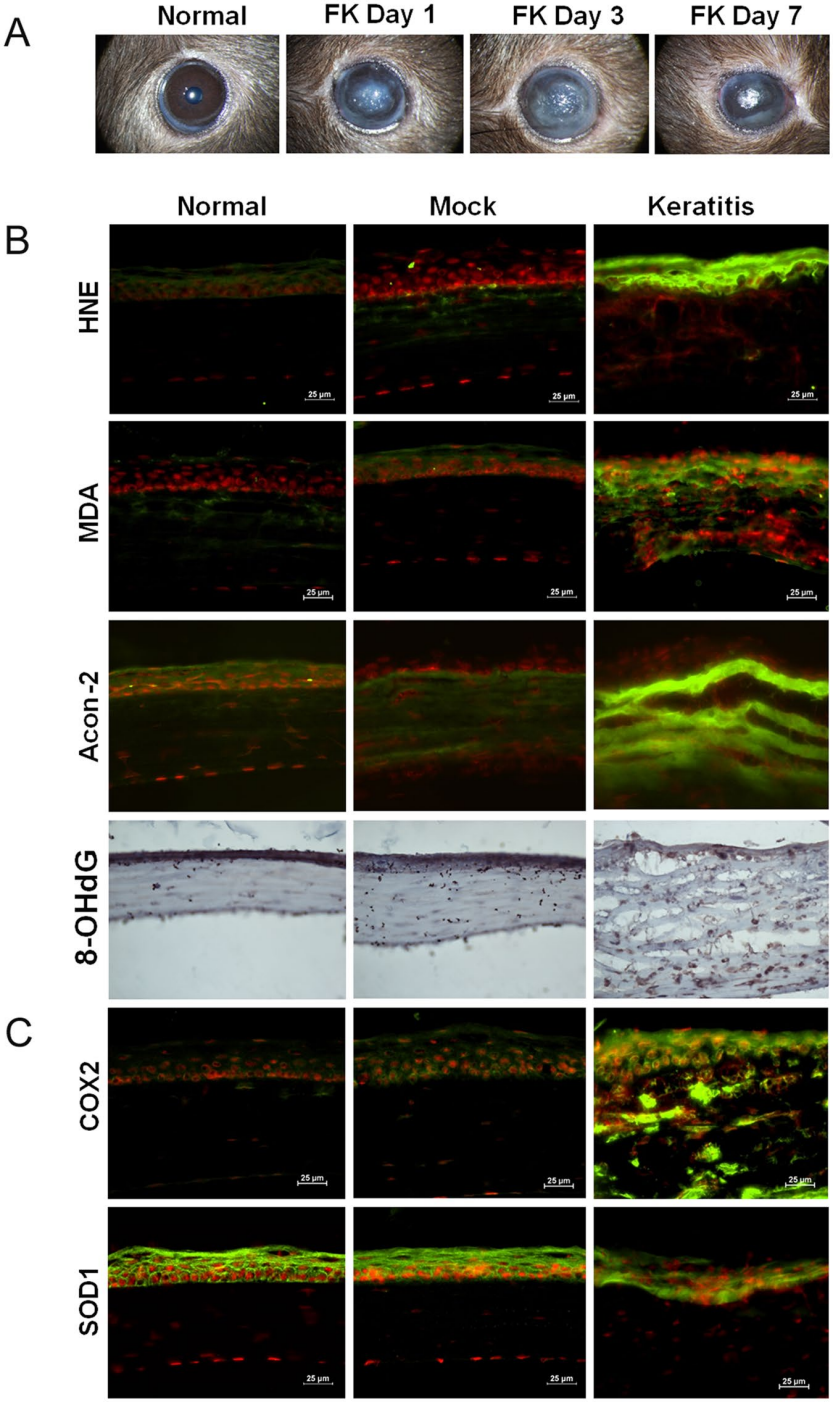
Murine model of fungal keratitis with the expression of oxidative stress biomarkers *in situ*. (**A**) Clinical signs of fungal keratitis in the murine model on day 1, day 3, and day 7 p.i. with *C. albicans* compared with a mock control treated with PBS. (**B**) Protein expression of biomarkers of oxidative stress in tissue with keratomycosis and mock controls. (**C**) Protein expression of oxidative stress-related enzymes in tissue with keratomycosis and mock controls.

An experimental mouse model of fungal keratitis was created by inoculation with 1 × 10^6^ colony-forming units (CFU) of *C. albicans* after the cornea in the right eye of C57BL/6 J mice were scarified with a 22-gauge needle. All eyes inoculated with *C. albicans* showed clinical signs of fungal keratitis, including corneal edema and opacity with stromal infiltration, as examined using a slit-lamp microscope. Corneal inflammation started on day 1 post-inoculation (p.i.) (mean score 7.2 ± 0.5), peaked on day 3 p.i. (8.1 ± 0.7), and then decreased on day 7 p.i. (6.3 ± 0.9) (Fig. 1A). No corneal infection was observed in the eyes of phosphate-buffered saline (PBS)-inoculated mice, which served as mock controls.

### Altered expression profile of oxidative stress-related genes in mouse corneas with fungal keratitis

Affymetrix microarray analysis revealed differential expression of oxidative stress related genes between *C. albicans*-infected corneas and PBS mock controls. As shown in Table 1, the average ratios of the expression levels of the Hmox1, Nos3, Hif1a, Sod2, Sod3, and Gpx2 genes increased more than 2-fold (p < 0.05) at day 1 p.i. in the fungal keratitis model, whereas the expression ratios of the Hif1an and Prdx6 genes decreased more than 2-fold (p < 0.05).

**Table 1:** Expression ratios of oxidative stress-related genes in the corneas of mice with *C. albicans*-induced keratitis and PBS mock controls.

Symbol	Ratio	P	Accession	Description
Hmox1	20.1	2.2E-03	451524	Heme oxygenase (decycling) 1
Cox2	49.9	5.0E-03	436280	Cyclooxygenase 2
Nos3	2.3	2.1E-02	441804	Nitric oxide synthase 3, endothelial cell
Hif1a	3.0	2.5E-02	438856	Hypoxia inducible factor 1, alpha subunit
Sod1	1.2	4.6E-01	439289	Superoxide dismutase 1, mitochondrial
Sod2	2.6	1.6E-02	447652	Superoxide dismutase 2, mitochondrial
Sod3	2.3	2.0E-02	432340	Superoxide dismutase 3, extracellular
Gpx1	−1.1	1.9E-01	427284	Glutathione peroxidase 1
Gpx2	2.8	1.6E-02	452576	Glutathione peroxidase 2
Hif1an	−2.0	8.4E-03	442831	Hif1a inhibitor
Prdx2	−1.5	3.2E-02	446030	Peroxiredoxin 2
Prdx6	−2.9	2.8E-03	457206	Peroxiredoxin 6

### Corneal oxidative damage in experimental fungal keratitis

Lipid peroxidation of the cell membrane is one of the major consequences of ROS overproduction, leading to production of conjugated diene hydroperoxides and unstable substances that disintegrate into various toxic aldehydes, such as 4-hydroxynonenal (4-HNE) and malondialdehyde (MDA); these are biomarkers of oxidative damage via lipid membrane peroxidation. As evaluated by immunofluorescent staining, production of 4-HNE and MDA was largely induced in the corneas with keratomycosis compared with mock controls or untreated normal corneas (Fig. 1B). Aconitase-2, one of the enzymes participating in the tricarboxylic acid cycle, acts as a biosensor for oxidative stress and protects mitochondrial DNA from oxidative damage, and in our study, aconitase-2 protein levels were found to be markedly increased in the corneas with keratomycosis based on immunofluorescent staining (Fig. 1B). At the same time, the oxidative DNA damage biomarker 8-OHdG was highly expressed in the corneal tissue of the mice with *C. albicans*-induced keratitis (Fig. 1B). Furthermore, the oxidative enzyme cyclooxygenase-2 (COX2) was barely expressed in normal and mock control corneas, whereas its expression was induced dramatically in the corneal epithelium as well as in stromal cells in fungal keratitis (Fig. 1C). Interestingly, the antioxidant enzyme superoxide dismutase-1 (SOD1) was strongly produced in normal and mock control corneas, but its levels decreased significantly in fungal keratitis (Fig. 1C).

### Increased ROS production in primary HCECs exposed to *C. albicans*

**Figure 2:**
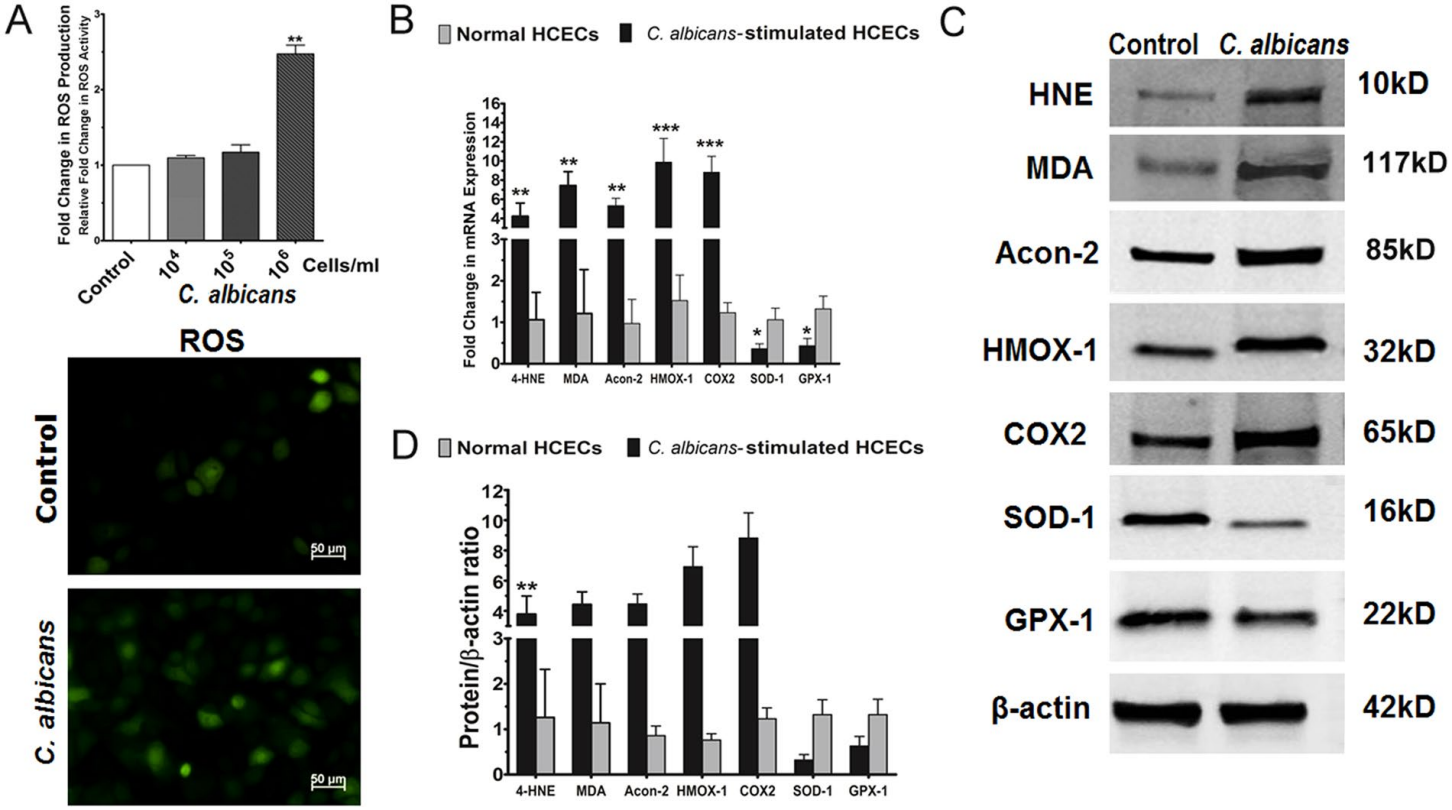
Increased ROS production and expression of oxidative stress biomarkers in primary HCECs exposed to *C. albicans*. (**A**) Increased ROS production in primary cultured HCECs challenged with different doses of *C. albicans* (0, 10^4^, 10^5^, or 10^6^ CFU) for 2 hours, as measured by fluorescence spectroscopy and microscopy. (**B**) Gene expression of oxidative stress biomarkers in HCECs challenged with *C. albicans*, as assessed by real-time PCR. (**C**) Protein expression of oxidative stress biomarkers in HCECs challenged with *C. albicans*, as assessed by Western blot analysis. (**D**) Protein/β-actin ratio for oxidative stress biomarkers in HCECs challenged with *C. albicans*.

To confirm the oxidative damage observed in fungal keratitis, an *in vitro* culture model was created by challenging primary HCECs with different doses of *C. albicans* (0, 10^4^, 10^5^, or 10^6^ CFU) for 2 hours. Cellular ROS production in the HCECs was then measured using a DCFDA kit along with both fluorescence spectroscopy and microscopy. As shown in Fig. 2A, ROS production increased in HCECs exposed to higher doses of *C. albicans*. In particular, ROS fluorescence intensity increased 2.5-fold (p < 0.01) in HCECs exposed 10^6^ CFU of *C. albicans* compared with untreated controls. Moreover, DCF fluorescence microscopy clearly showed many more positive cells with stronger fluorescent intensity in *C. albicans*-challenged HCECs than in the untreated controls.

### Biomarkers of oxidative injury in HCECs challenged with *C. albicans*

The gene and protein expression of oxidative biomarkers was evaluated by real-time reverse transcription-polymerase chain reaction (RT-PCR) and Western blot analysis (Fig. 2B–D). The cell membrane lipid peroxidation markers HNE and MDA were respectively overproduced by 4.22 ± 1.38-fold and 7.43 ± 1.46-fold at the gene level and 4.79 ± 1.43-fold and 6.43 ± 0.83-fold at the protein level in HCECs exposed to 1 × 10^6^ CFU of *C. albicans*. Aconitase-2 mRNA and protein levels increased significantly, by 5.33 ± 0.78-fold and 7.46 ± 1.66-fold, respectively. Immunofluorescent staining further confirmed that the levels of these three markers increased in HCECs exposed to *C. albicans*. Interestingly, the oxidants heme oxygenase-1 (HMOX1) and COX2 were found to be respectively upregulated by 9.87 ± 2.50-fold and 8.82 ± 1.67-fold at the gene level and 6.79 ± 1.23-fold and 4.43 ± 1.23-fold at the protein level, whereas the antioxidative enzymes SOD1 and glutathione peroxidase-1 (GPX1) were respectively downregulated by 0.36 ± 0.12-fold and 0.43 ± 0.18-fold at the gene level and 0.46 ± 0.26-fold and 0.52 ± 0.17-fold at the protein level in HCECs challenged with *C. albicans*.

### Activation of p38 MAPK in experimental fungal keratitis and in HCECs exposed to *C. albicans*

**Figure 3:**
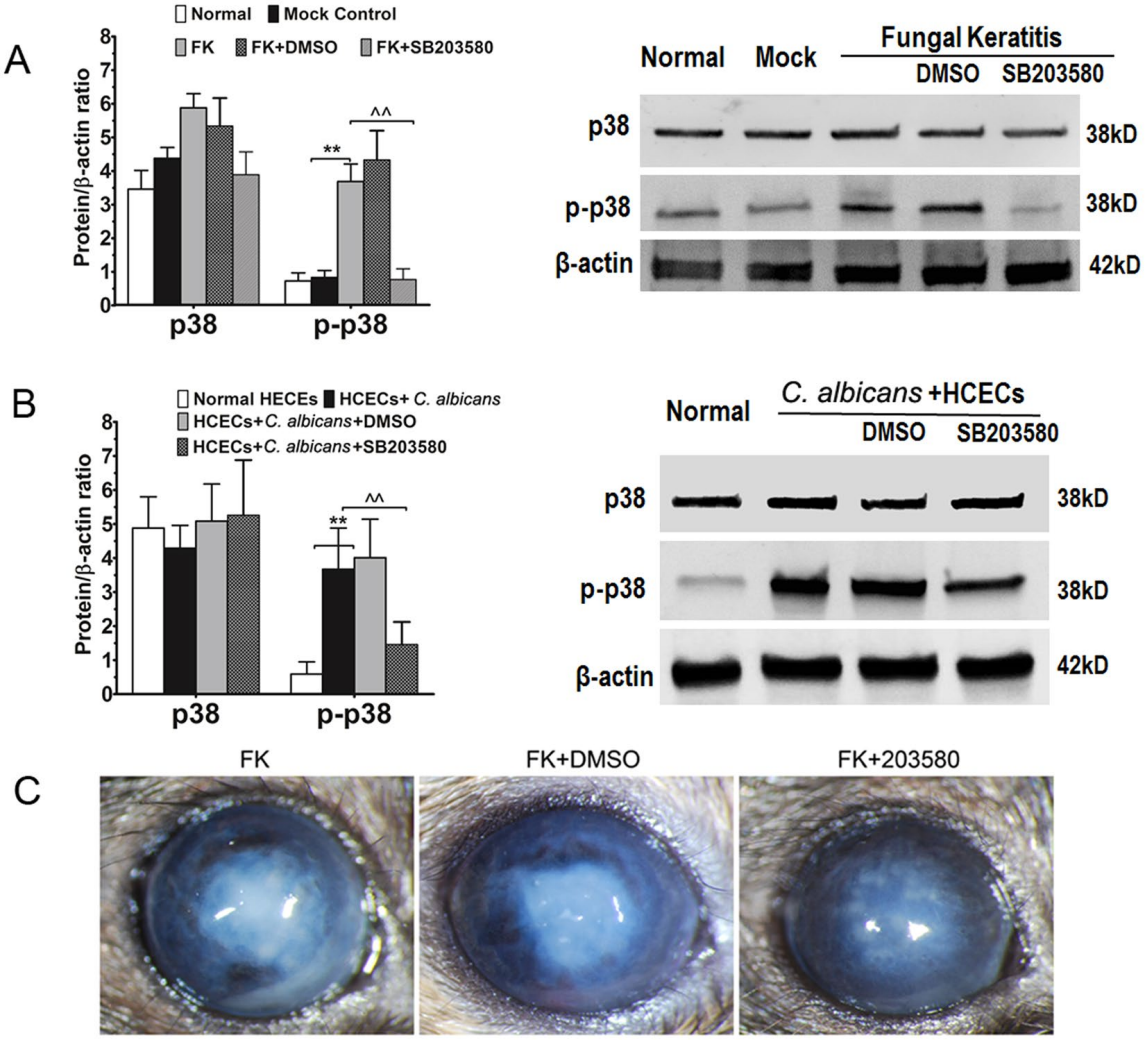
Activation of p38 MAPK in the murine model of fungal keratitis and in cultured HCECs challenged with *C. albicans*. (**A**) Increased expression of p-p38 in tissue with keratomycosis compared with controls and inhibition by the p38 activation inhibitor SB203580. (**B**) Increased expression of p-p38 in HCECs challenged with *C. albicans* compared with controls and inhibition by the p38 activation inhibitor SB203580. (**C**) Fungal keratitis with SB203580 treatment showed much less opacity compared with DMSO treatment and FK control on Day 7 p.i.

Activation of p38 MAPK was evaluated in both the animal model and *in vitro* cultured HCECs by Western blot analysis of p38 protein phosphorylation. As shown in Fig. 3A, the protein level of phosphorylated p38 (p-p38) increased significantly (3.69 ± 0.52-fold, p < 0.01) in mouse corneas with fungal keratitis, but not in the corneas of PBS mock control mice. However, the total p38 level was not changed in corneas with different treatments compared with normal controls. Interestingly, the stimulated p-p38 level was largely suppressed (0.77 ± 0.32-fold, p < 0.01) in corneas with keratitis treated with the p38 activation inhibitor SB203580 1 hour prior, but not in DMSO-treated controls. This finding indicates that p38 MAPK was activated in the corneas of mice with *C. albicans*-induced keratitis.

p38 activation was confirmed in the HCEC culture model. As shown in Fig. 3B, p38 protein was phosphorylated and activated in HCECs stimulated by *C. albicans*, as demonstrated by upregulation of the p-p38 level (3.67 ± 1.21-fold, p < 0.01), but not total p38 production. The level of p-p38 was significantly blocked (1.46 ± 0.66-fold, p < 0.01) by SB203580 (1 μM in DMSO), which was added to the culture 1 hour prior to *C. albicans* exposure.

### p38 MAPK inhibitor reduced corneal opacification but not fungal load

Corneal opacity was monitored on day 1 to day 7 p.i. respectively, and scores were compared between the FK mice with or without p38 inhibitor SB203580, and DMSO subconjunctival injection. The mean score of corneal opacity in the group with SB203580 on day 1 p.i. (mean score 4.3 ± 0.5) was lower compared to the FK mice without SB203580 (5.8 ± 0.7, p < 0.05) or FK mice with DMSO injection (5.5 ± 0.3, p < 0.05), and on day 7 p.i. the SB203580 group showed less opacity (mean score 3.4 ± 0.5) than the FK mice without SB203580 (4.8 ± 0.7, p < 0.05) or DMSO injection (4.5 ± 0.3, p < 0.05) (Fig. 3C).

As to know whether p38 inhibitor SB203580 could kill or affect fungi growth, we performed recovery culture from infected cornea tissue on day 1 p.i. in each group. The number of colonies recovered from infected corneas with SB203580 (2643.0 ± 1772.1) and DMSO (2589.2 ± 1333.7) injection group were similar to those in FK mice without injection (2833.3 ± 1690.5).

### p38 MAPK pathway involved in oxidative injury in fungally challenged HCECs

**Figure 4:**
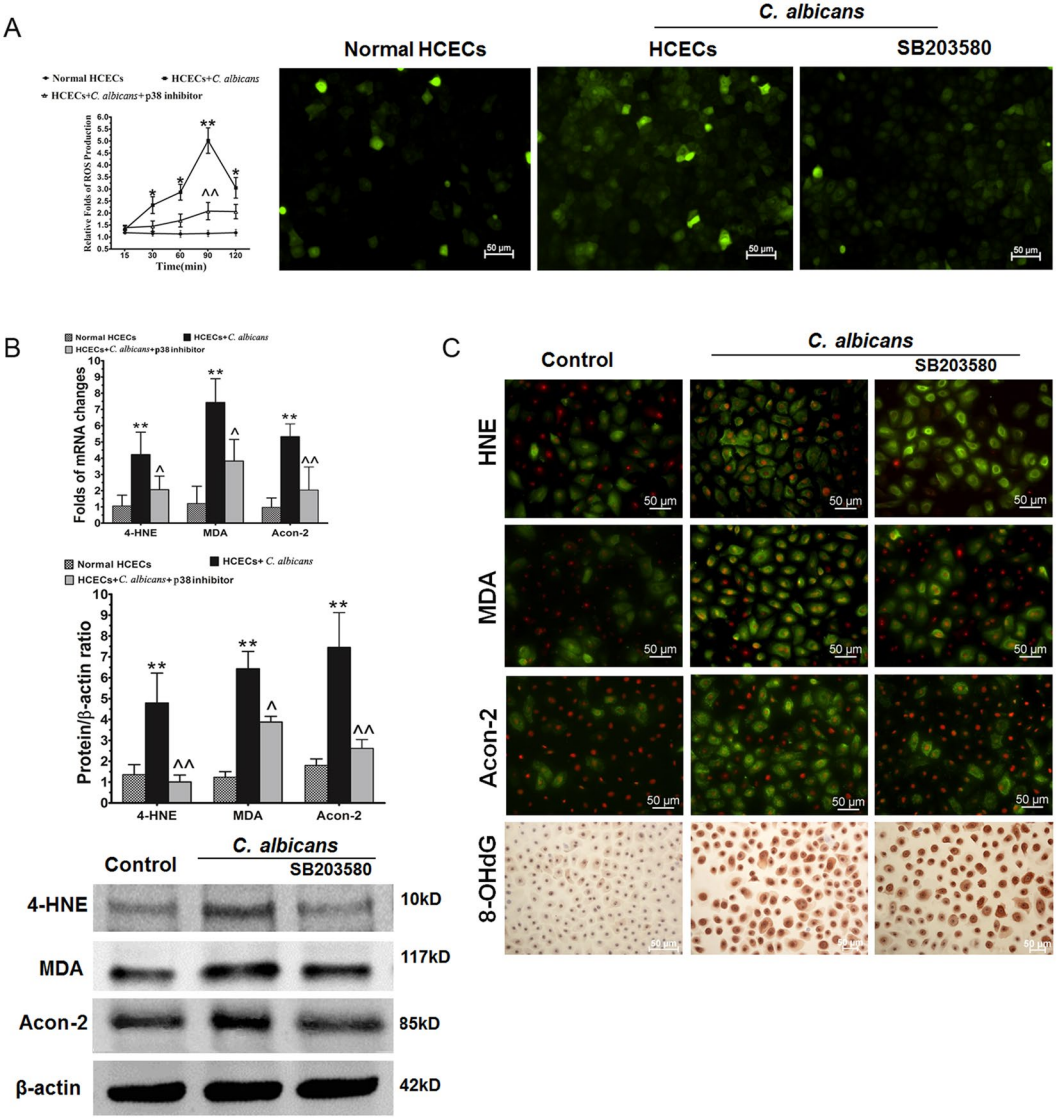
p38 MAPK pathway involved in oxidative injury to HCECs challenged with *C. albicans*. (**A**) Increased ROS generation in HCECs challenged with *C. albicans* and inhibition by the p38 activation inhibitor SB203580. (**B**) Gene and protein expression of oxidative stress biomarkers inhibited by SB203580 in HCECs challenged with *C. albicans*. (**C**) Increased *in situ* protein expression of oxidative stress markers in HCECs challenged with *C. albicans* following treatment with the inhibitor SB203580.

We then investigated the role of the p38 signaling pathway in ROS generation and oxidative injury in fungally challenged HCECs. As shown in Fig. 4A, ROS production started to increase at 30 minutes and reached a 5-fold peak level at 90 minutes in HCECs exposed to 1 × 10^6^ *C. albicans*. The increased production of ROS was largely suppressed to near-normal control levels (p < 0.01) by prior incubation with 1 µM SB203580. Significant increases in the DCF fluorescence intensity and the number of DCF-positive cells were also observed in HCECs exposed to *C. albicans* for 90 minutes compared with the normal control, as determined using fluorescence microscopy. However, the rise in DCF fluorescence was significantly blocked by incubation with 1 µM SB203580 1 hour prior.

With ROS generation suppressed, the levels of biomarkers of fungus-induced oxidative damage, including HNE, MDA, aconitase-2, and 8-OHdG, were also decreased significantly by the p38 inhibitor SB203580, as shown by real-time RT-PCR and Western blotting (Fig. 4A). Immunofluorescent and immunohistochemical staining further showed high immunoreactivity for HNE, MDA, and aconitase-2 in the cell membrane and cytoplasm and for 8-OHdG in the nuclear area in HCECs exposed to *C. albicans*. However, treatment with 1 µM SB203580 dramatically reduced the production of these biomarkers (Fig. 4B), indicating the critical role of p38 activation in fungal oxidative injury.

### p38 pathway regulated balance between oxidant and antioxidative enzymes in HCECs stimulated by *C. albicans*

**Figure 5:**
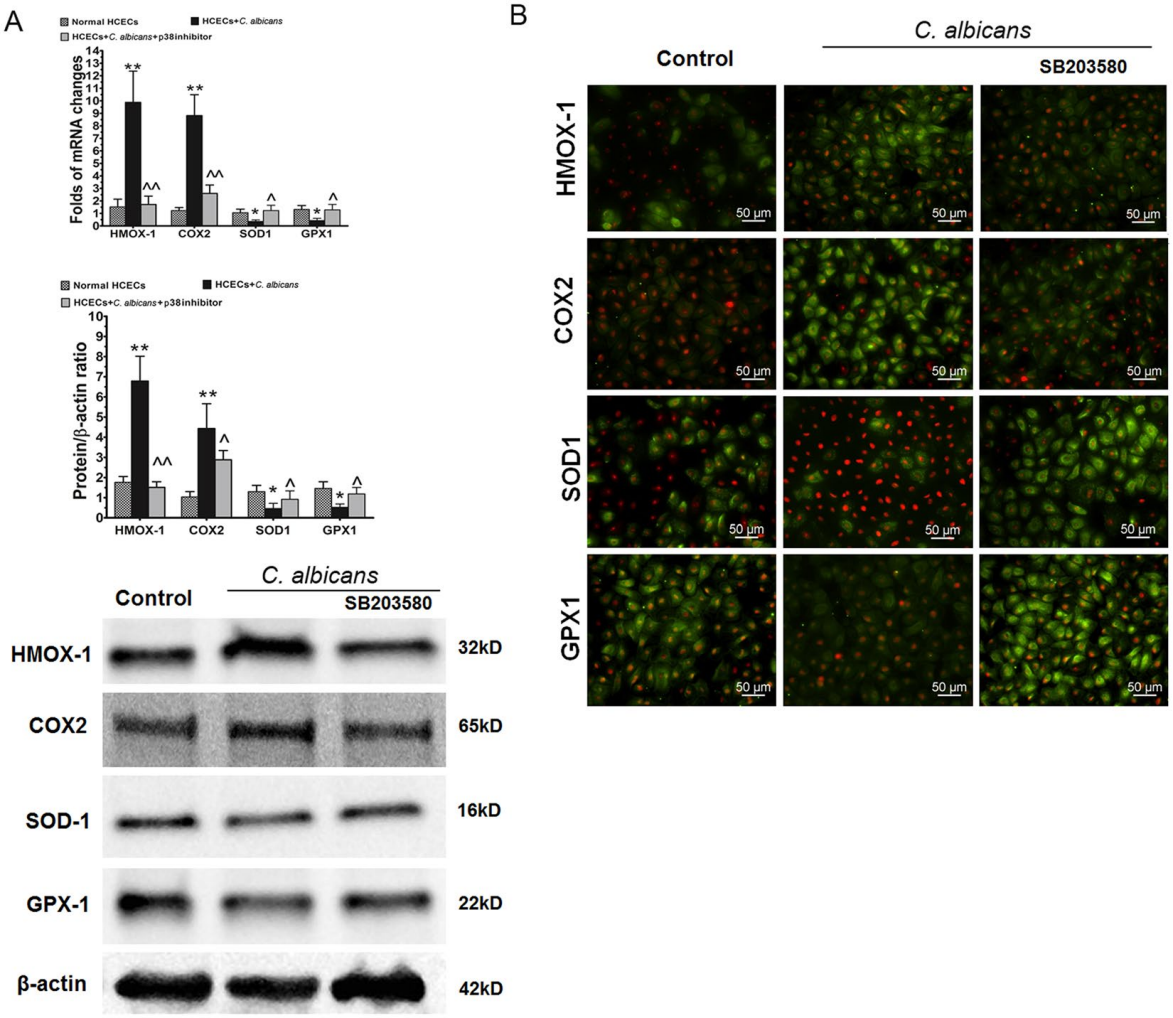
p38 MAPK pathway-mediated regulation of the balance of oxidant and antioxidative enzymes in HCECs challenged with *C. albicans*. (**A**) Gene and protein expression of oxidant and antioxidative enzymes in HCECs challenged with *C. albicans* with or without SB203580 treatment. (**B**) *In situ* protein expression of oxidant and antioxidative enzymes in HCECs challenged with *C. albicans* with or without SB203580 treatment.

As shown in Fig. 5A, production of the oxygenases HMOX1 and COX2 increased as the expression of the antioxidative enzymes SOD1 and GPX1 decreased in HCECs challenged with *C. albicans*, as evaluated on both the gene and protein expression levels by RT-PCR and Western blotting, respectively. However, the p38 inhibitor SB203580 suppressed the expression of HMOX1 (1.71 ± 0.67-fold, p < 0.01) and COX2 (2.60 ± 0.67-fold, p < 0.01) but increased the levels of SOD1 (1.23 ± 0.42-fold, p < 0.05) and GPX1 (1.28 ± 0.44-fold, p < 0.05), which partially restored the imbalance caused in the HCECs by fungal exposure. Immunofluorescent staining confirmed these results (Fig. 5B). Additionally, SB203580 (1 µM) pretreatment suppressed the stimulated immunoreactivity for HMOX1 and COX2 but increased the expression of the antioxidative enzymes SOD1 and GPX1 in fungally challenged HCECs.

## Discussion

Oxidative stress results from disturbances in the prooxidative/antioxidative cellular balance due to elevated levels of ROS-producing oxidation reactions. ROS play an important role in the regulation of many physiological processes involved in intracellular signaling^22^, but they can also induce serious damage to biomolecules. A disturbance in the balance between free-radical-generating and radical-scavenging systems results in oxidative stress, which is associated with cellular damage in many pathological conditions and diseases^23–25^. Oxidative stress has specifically been found to occur in non-infective diseases, such as keratoconus, bullous keratopathy, and Fuchs endothelial dystrophy^26^. In infectious keratitis, neutrophils are the predominant infiltrating cell type, and they induce tissue destruction by releasing proteolytic enzymes, ROS, and reactive nitrogen species^27, 28^. Moreover, proinflammatory cytokines released by neutrophils in the inflamed cornea may promote the release of ROS, with a consequent increase in oxidative damage^27, 29^. The present study showed that oxidative stress is involved in *C. albicans*-induced fungal keratitis *in vivo* as well as in HCECs challenged with *C. albicans in vitro*.

Lipid peroxidation (autoxidation) is a process of oxidation of polyunsaturated fatty acids due to the presence of several double bonds in their structure, and it involves production of peroxides and reactive organic free radicals. MDA, a toxic aldehyde resulting from membrane lipid peroxidation, and 4-HNE, an α,β-unsaturated hydroxyalkenal, have been identified as the most cytotoxic breakdown products generated from lipid peroxidation^30–32^. ROS also attack structural and enzymatic proteins by oxidizing residual amino acids and prosthetic groups, forming crosslinks, and inducing protein aggregates as well as proteolysis. Meanwhile, aconitase-2 is an iron-sulfur protein, or nonheme iron protein, and its iron-sulfur clusters are thought to play a role in the electron-transfer reactions of oxidative phosphorylation^33^. Aconitase-2 levels thus increase with increased mitochondrial activity in cells under oxidative stress^34, 35^. ROS can also react with nucleic acids, attacking the nitrogenous bases and the sugar phosphate backbone. Furthermore, ROS can induce single- and double-stranded DNA breaks. Finally, 8-OHdG is a product of oxidative DNA damage due to specific enzymatic cleavage, namely, ROS-induced 8-hydroxylation of the guanine base in mitochondrial and nuclear DNA^36^. Therefore, 8-OHdG release has been widely used as a sensitive and reliable marker of oxidative DNA damage^37^. In the present study, the protein levels of MDA, 4-HNE, aconitase-2, and 8-OHdG were upregulated in corneal tissue with fungal infection, which indicated that oxidative stress occurred in fungal keratitis and caused the corneal tissue, and especially corneal epithelial cells, to undergo oxidative injury, including via lipid peroxidation, intracellular protein modification, and DNA oxidative damage. All of this evidence showed that oxidative stress-related processes occur during the early stage of fungal keratitis, given that the expression of all these key markers and molecules increased dramatically in infected tissues compared with the tissues of normal controls.

COX2 is a major oxygenase that is highly induced by a variety of agents, causing oxidative stress, and upregulation of COX is a common feature of inflammation resulting from oxidative stress. In particular, COX2, officially known as prostaglandin endoperoxide synthase, is an enzyme that mediates oxidative stress^38, 39^. Antioxidant enzymes form the first line of defense against free radicals and toxic reactants in organisms, metabolizing them into innocuous byproducts^40^. In turn, oxidative stress has been speculated to cause antioxidant consumption, resulting in a decline in the antioxidant level^41^. Thus, the levels of antioxidant enzymes may vary during oxidative stress, given that antioxidant enzymes such as glutathione peroxidase (GPX) and superoxide dismutase (SOD) may be induced by oxidative stress (to increase their level or activity) or consumed during this stress (to decrease their level or activity)^42^. The activities of SOD isoenzymes have been specifically observed in tears, the cornea, and other parts of the human eye^43^. Our findings revealed that fungal infection interrupted the antioxidant defense system by increasing the level of the oxygenase COX2 and reducing the level of the antioxidant enzyme SOD1, which caused oxidative stress in inflamed corneal tissue.

*C. albicans* is a fungus commonly found in the human mucosa that may cause superficial and systemic infections, especially in immunosuppressed individuals. Until now, the main actors in the defense against this fungus were believed to be epithelial cells, neutrophils, macrophages/monocytes and dendritic cells. In particular, inflammatory cells are known to be involved in phagocytosis and ROS production following challenge with *C. albicans*. However, corneal epithelial cells are located in such a way as to serve as the first line of anti-Candida defense. Our results showed that corneal epithelial cells produce ROS during stimulation by *C. albicans* and that this activity may contribute substantially to the fungicidal process. Under physiological conditions, ROS have bactericidal effects, participating in corneal defense. However, oxidative stress derived from excessive ROS production that surpasses the buffering capability of antioxidant defense in the host causes oxidative injury to the corneal epithelial cells as well as ocular mucosal barrier damage, microorganism translocation, and an inflammatory response^44, 45^.

ROS are highly active chemical forms that target the components of corneal epithelial cells, such as proteins, lipids, and nucleic acids, leading to lipid peroxidation, protein dysfunction, and DNA mutations, respectively. In the present study, biomarkers of lipid peroxidation, including MDA and HNE, were upregulated in corneal epithelial cells stimulated by *C. albicans*. Lipid peroxides are important secondary injury factors contributing to oxidative injury, as confirmed by the increase in biomarkers of oxidative stress in protein and DNA, that is, aconitase-2 and 8-OHdG, respectively, in HCECs in the current study. Protein adducts modified by aconitase-2 can also act as special second messengers or autoantigens, promoting macrophage accumulation, retention, and activation and thus increasing ROS generation. 8-OHdG is a predominant form and is a valuable biomarker widely used to assess endogenous oxidative damage to DNA.

Antioxidant enzymes form the first line of defense against free radicals and toxic reactants in organisms, metabolizing them into innocuous byproducts^40^. Three categories of important enzymes, or SOD, GPX, and PRDX, were investigated in the corneal tissue and HCECs in the present study. Our findings showed that SOD2, SOD3, and GPX2 were upregulated at the mRNA level in corneal tissues and that SOD1, GPX1, and PRDX4 were upregulated at the protein level in cells. All of these are important molecules in biological oxidation in corneal epithelial cells and participate in reducing H_2_O_2_ to H_2_O and clearing free radicals. This evidence is consistent with the finding that corneal epithelial cells have evolved an antioxidant defense system to maintain homeostasis between oxidant and antioxidant species^46, 47^.

The MAPK signaling pathways contain highly conserved serine/threonine protein kinases that function in various fundamental cellular processes, such as growth/proliferation, differentiation, motility, and apoptosis/survival as well as the stress response and the response to infection. Several of the MAPK family members have been shown to be involved in corneal inflammation caused by adenoviruses^48^ and staphylococci^49^ and in certain pathological processes progressing from the corneal epithelium to the endothelium^49–51^. Although patients with FK have been treated with antifungal drugs that prevent the progression of corneal pathogenesis, further inflammatory reaction to the fungus still destroy the corneal tissue. FK-induced corneal inflammation is characterized by increased corneal thickness and haze and neutrophil recruitment to corneal stroma. Our findings suggested that the p38 MAPK pathway may play certain biological roles during fungal infection of the cornea, especially in corneal epithelial cells exposed to *C. albicans*, given that during this process, the pathway was activated and downstream molecules were upregulated. Therefore the inhibition of p38 activation may reduce the corneal inflammation, and opacification for some extent. It is reported that SB203580 had been used in some murine models of a broad range of disorders, including inflammation with arthritis. Of particular interest for fungal keratitis, the selective use of p38 inhibitor in anti-inflammatory combined with antimicrobial activity could be a novel auxiliary treatment strategy^52^.

Oxidative stress may also activate MAPK signaling pathways. Increased ROS production can specifically activate ERKs, JNKs or p38 MAPKs, as reported under different conditions^53, 54^. The exact mechanism by which ROS activate these kinases is unclear, but a possible mechanism may be related to oxidative modifications and resultant activation of the signaling effector proteins and inactivation and/or degradation of MAPK phosphatases^55^. However, certain studies have shown that an inhibitor of p38 MAPK could decrease the ROS level in immortalized epithelial cells^56^ and adult stem cells^57^. Interestingly, we found that the inhibitor of p-p38 SB203580 could suppress both the activation of p38 MAPK and ROS production to a certain extent in HCECs stimulated by HKCA due to downregulation of the gene and protein expression of HMOX1 and PTGS2; this finding suggested that the p38 MAPK pathway plays certain roles in oxidative stress in HCECs and that pharmaceutical inhibition of p38 may reduce ROS-mediated damage to fungally infected corneas. Our findings also suggest that blocking the activity of p38 MAPK may reduce ROS-mediated damage to the tissues, which is a complementary effect of the multifunctionality of p38 MAPK, which is involved in both physiological and pathological processes.

In conclusion, during fungal infection in corneal epithelial cells, the balance of oxidation and antioxidation is disturbed, and the resultant oxidative stress is involved in the development of fungal keratitis. In this context, inhibition of p38 MAPK may decrease ROS production and consequently decrease the damage caused by oxidative stress.

## Materials and Methods

### *C. albicans* and mouse model of fungal keratitis

A clinically isolated strain of *C. albicans*, SC5314, was cultured on Sabouraud dextrose agar (Difco, Detroit, MI) for 3 days at 25 °C and then harvested and diluted in sterile PBS to yield a 1 × 10^6^ CFU/5 µL inoculum based on the optical density (OD), with an OD_600_ conversion factor of 3 × 10^7^ CFU/mL^58^. Experimental animals were treated in accordance with the ARVO Statement for the Use of Animals in Ophthalmic and Vision Research, and the protocols were approved by the Tianjin Medical University Institutional Animal Care and Use Committee. In this study, 6- to 8-week-old female C57BL/6 J mice were anesthetized by intraperitoneal injection of ketamine and xylazine. The right corneas of the mice were then scarified with a 22-gauge needle, followed by inoculation with 1 × 10^6^ CFU of *C. albicans* or with sterile PBS to induce keratitis or establish a mock control, respectively. The mice were then monitored daily for one week p.i. under a dissecting microscope to evaluate corneal inflammation; the grading system included the relative infiltration area, opacity density, and surface regularity of the corneas^59^.

### *In vitro* culture model of HCECs challenged with *C. albicans*

Human donor corneoscleral tissues that did not meet the criteria for clinical use were obtained from Tianjin Eye Hospital (Tianjin, China). These human tissues were handled according to the tenets of the Declaration of Helsinki. In brief, each limbal rim was dissected into 12 explants. Each piece was then placed in a supplemented hormonal epidermal medium containing 5% fetal bovine serum, followed by incubation at 37 °C under 5% CO_2_ and 95% humidity. Corneal epithelial cell growth was carefully monitored until the confluent stage. After that, the cultured corneal epithelial cells were switched to serum-free supplemented hormonal epidermal medium for 24 hours and then exposed to *C. albicans* for different time periods (4, 24 or 48 hours)^60^.

### Cellular ROS production assay

Cellular ROS production was measured using a DCFDA assay kit (Abcam, Cambridge, MA) according to the manufacturer’s protocol. DCFDA (2′,7′-dichlorofluorescein diacetate), a cell-permeable fluorogenic dye, is deacetylated to a non-fluorescent compound by cellular esterases and later oxidized into highly fluorescent DCF (2′,7′-dichlorofluorescein) by ROS, allowing measurement of hydroxyl, peroxyl and other ROS activity within the cell. Here, HCECs were grown on 96-well plates or 8-chamber slides. Once they reached confluence, the cells were washed twice with PBS and then incubated with 25 μM DCFDA in essential medium with 10% FBS in a 37 °C incubator for 45 minutes. After two washes with PBS, the cells were exposed to different concentrations of *C. albicans* (0, 10^4^, 10^5^, or 10^6^ CFU). Images of the cells were then taken under a fluorescence microscope. In addition, cell fluorescence in the 96-well plates was measured at 488 nm excitation and 525 nm emission using a Tecan Infinite M200 Multimode Microplate Reader (Tecan US, Inc. Morrisville, NC) after adding *C. albicans* for 15–120 minutes. Relative changes in DCF fluorescence are expressed as fold increases over fluorescence in untreated cells.

### Affymetrix microarray analysis of mouse corneas with *C. albicans*-induced keratitis

Microarray analysis was performed according to previously published protocols^61^. Briefly, after quality assurance of RNA samples from corneas with fungal keratitis and mock controls, two cycles of amplification (Affymetrix GeneChip 430.2; Affymetrix, Santa Clara, CA) were applied to qualified samples from 3 five-cornea pools from each group. Images and quality-control metrics were recorded using Affymetrix GCOS software version 1.4, and signal-intensity data were adjusted and analyzed with BioConductor software. The criteria for significant changes in gene expression were established as >2-fold and adjusted p < 0.05.

### Total RNA extraction, reverse transcription (RT) and real-time quantitative PCR (qPCR)

Mouse corneas were carefully dissected from the surrounding conjunctiva and uvea after scarification. Pools of 5 corneas were then prepared in triplicate from *C. albicans*-infected and control groups at day 1 p.i., followed by lysis in RLT buffer. After HCECs were exposed to different treatments, the cells were also lysed in RLT buffer. Total RNA was extracted with an RNeasy Plus Mini Kit (Qiagen, Valencia, CA) for the primary HCECs and with an RNeasy Plus Micro Kit (Qiagen, Valencia, CA) for the mouse corneas according to the manufacturer’s protocols and was quantified with a spectrophotometer (NanoDrop ND-1000; Thermo Scientific, Wilmington, DE) and stored at −80 °C before use. Subsequently, first-strand cDNA was synthesized from 1.0 µg of total RNA (Ready-To-Go You-Prime First-Strand Beads, GE Healthcare, Princeton, NJ) and random hexamers (Applied Biosystems, Foster City, CA). Real-time RT-PCR was then performed using an Mx3005 P QPCR System (Stratagene, La Jolla, CA). The TaqMan gene expression assays used in this study were specific for 4-HNE, MDA, aconitase-2, HMOX1, COX2, GPX2, and SOD1. The threshold cycle (C_T_) for each target mRNA was normalized to glyceraldehyde-3-phosphate dehydrogenase (GAPDH) mRNA and averaged. Two-group comparisons were performed using Student’s t-test, and three-group comparisons were conducted using one-way analysis of variance (ANOVA). A value of p < 0.05 was considered statistically significant.

### Immunofluorescent and immunohistochemical staining

Mouse corneas obtained on day 1 p.i. were embedded in OCT compound (Sakura Finetek, Torrance, CA), snap-frozen in liquid nitrogen, and sectioned at 10 µm thickness. The sections were then thawed, dehydrated, and fixed in 4% paraformaldehyde for 10 minutes, followed by treatment with 0.3% hydrogen peroxide and 10% normal donkey serum. After primary antibodies were applied (1:200), biotin-conjugated donkey anti-rabbit or anti-goat secondary antibodies (Jackson ImmunoResearch Laboratories, West Grove, PA) were applied, followed by processing with a Vectastain Elite ABC Kit (Vector Laboratories, Burlingame, CA). The sections were then incubated with diaminobenzidine (DAB) as a chromogen, followed by counterstaining with hematoxylin. Finally, the sections were dehydrated, mounted with a coverslip, and photographed with an epifluorescence microscope.

The HCECs cultured on 8-chamber slides were fixed in freshly prepared 2% paraformaldehyde at 4 °C for 10 minutes, followed by permeabilization with 0.2% Triton X-100 in PBS at room temperature for 10 minutes. Indirect immunofluorescent staining was performed for sections and cells using our previous methods^58, 62^. Antibodies against MDA, HNE, aconitase-2, GPX1 and PDRX4 purchased from Abcam (Cambridge, CA) and antibodies against 8-OHdG, HMOX1, COX2, and SOD1 purchased from Santa Cruz Biotechnology (Santa Cruz, CA) were applied as primary antibodies. Alexa-Fluor 488-conjugated secondary antibodies (donkey anti-goat IgG, donkey anti-rabbit IgG, or goat anti-mouse IgG) from Molecular Probes (Eugene, OR, http://www.invitrogen.com) were subsequently applied, and propidium iodide (PI) was used for nuclear counterstaining for immunofluorescent staining. The stained slides were photographed with a Zeiss laser scanning confocal microscope (LSCM510META, Thornwood, NY).

### Western blot analysis

Western blot analysis was performed using a previously reported method^63^. In brief, cytoplasmic and nuclear extracts were prepared using a Nuclear Extract Kit (Active Motif) according to the manufacturer’s instructions. Equal amounts of protein, as measured using a BCA protein assay kit, were mixed with 6 × SDS reducing sample buffer and boiled for 10 minutes before loading. The proteins (50 µg/lane) were then separated on an SDS polyacrylamide gel and transferred electronically to PVDF membranes. After the membranes were blocked with 5% nonfat milk in TTBS (50 mM Tris [pH 7.5], 0.9% NaCl, and 0.1% Tween-20) for 1 hour at room temperature, they were incubated with primary antibodies against MDA (1:200), 4-HNE (1:200), aconitase-2 (1:200), HMOX1 (1:200), COX2 (1:200), SOD1 (1:200), GPX1 (1:200), p38 (1:200) or p-p38 (1:200) (both from Abcam, Cambridge, CA), or β-actin (1:1000) (Santa Cruz Biotechnology, Santa Cruz, CA) overnight at 4 °C. After three washes with Tris-buffered saline with 0.05% Tween 20 for 10 minutes each, the membranes were incubated with HRP-conjugated goat anti-mouse IgG (1:1000) or goat anti-rabbit IgG (1:1000) for 1 hour at room temperature. The signals were then detected with a chemiluminescence reagent (ECL; GE Healthcare), and the images were acquired using an imaging station (model 2000R; Eastman Kodak, Rochester, NY).

### p38 MAPK signal pathway assay

Activation of p38 MAPK was evaluated in both the *in vitro* cultured HCECs and animal model. HCECs were pre-incubated with 1 μl p38 activation inhibitor SB203580 (1 μM in DMSO, Sigma-Aldrich, St Louis, MO), or 1 μl DMSO as a control, for 1 hour before addition of 10^6^ cells/ml HKCA and incubated for 4 and 24 hours, respectively, for total RNA extraction and protein collection. 5 μl p38 activation inhibitor SB203580 or DMSO was subconjunctival injection 2 hours prior to the mice cornea were inoculated with with 1 × 10^6^ CFU of *C. albicans*. Corneal edema and opacity were observed using slit lamp everyday p.i. for a week and corneal opacity was scored using a grading scale of 0 to 4: grade 0 = completely clear; grade 1 = slightly hazy, iris and pupils easily visible; grade 2 = slightly opaque, iris and pupils still detectable; grade 3 = opaque, pupils hardly detectable, and grade 4 = completely opaque with no view of the pupils^64^.

### Corneal fungal recovery culture

Five infected corneal from FK mice were excised at day 1 p.i. Serial dilutions were inoculated onto Sabouraud dextrose agar plates to estimate fungal recovery. Colony counts, converted in to CFU per cornea, were compared among different groups^65^.

### Statistical analysis

Student’s t-test was used to examine differences between two groups. One-way ANOVA was used to perform comparisons among three or more groups, followed by Dunnett’s post hoc test. A value of p < 0.05 was considered statistically significant.
